# Comparison of patient-reported outcomes measurement information system (PROMIS®)-29 and PROMIS global physical and mental health scores

**DOI:** 10.1007/s11136-023-03559-y

**Published:** 2023-12-27

**Authors:** Ron D. Hays, Patricia M. Herman, Anthony Rodriguez, Maria Orlando Edelen

**Affiliations:** 1 Division of General Internal Medicine and Health Services Research, Department of Medicine, University of California, 1100 Glendon Avenue Suite 850, Los Angeles, CA USA; 2 RAND Corporation, Behavioral and Policy Sciences, 1776 Main Street, Santa Monica, CA USA; 3 RAND Corporation, Behavioral and Policy Sciences, 20 Park Plaza #920, Boston, MA USA; 4 Department of Surgery, Brigham and Women’s Hospital, Patient Reported Outcomes, Value and Experience (PROVE) Center, Boston, MA USA

**Keywords:** Physical health, Mental health, Convergent validity, PROMIS®

## Abstract

**Purpose:**

The Patient-Reported Outcomes Measurement and Information System (PROMIS®): includes the PROMIS-29 physical and mental health summary and the PROMIS global physical and mental health scores. It is unknown how these scores coincide with one another. This study examines whether the scores yield similar or different information.

**Methods:**

The PROMIS-29 and the PROMIS global health items were administered to 5804 adults from Amazon’s Mechanical Turk (MTurk) in 2021–2022 and to 4060 adults in the Ipsos KnowledgePanel (KP) in 2022.

**Results:**

The median age of those in MTurk (KP) was 36 (54) and 53% (50%) were male. Mean T-scores on the PROMIS-29 and PROMIS global physical health scales were similar, but PROMIS global mental health was 3–4 points lower than the PROMIS-29 mental health summary score. Product-moment correlations ranged from 0.69 to 0.81 between the PROMIS-29 physical health and PROMIS global physical health scales and 0.56–0.69 between the mental health scales. Multi-trait multimethod analyses indicated that only a small proportion of the correlations between the two methods of measuring mental health were significantly more highly correlated with one another than correlations between physical and mental health.

**Conclusions:**

PROMIS-29 and PROMIS global mental health scales provide different information and, therefore, study conclusions may vary depending on which measure is used. Interpretation of results needs to consider that the PROMIS-29 mental health scale is a weighted combination of specific domains while the PROMIS global mental health scale is based on general mental health perceptions. Further comparisons of methods of assessing mental health are needed.

**Supplementary Information:**

The online version contains supplementary material available at 10.1007/s11136-023-03559-y.

## Plain English summary 

It is unknown if the Patient-Reported Outcomes Measurement and Information System (PROMIS®)-29 physical and mental health summary and the PROMIS global physical and mental health scores yield similar or different information. This study compares the two types of PROMIS physical and mental health scores in two large samples (5,804 adults from Amazon’s Mechanical Turk and 4060 adults in the Ipsos KnowledgePanel). The physical health scores were similar but the mental health scores differed. Conclusions can vary depending on which mental health measure is used.

## Introduction

Three-quarters of a century ago, the World Health Organization (WHO) proposed that health consists of physical, mental, and social well-being [1]. Consistent with that, health-related quality (HRQOL) includes physical, mental, and social functioning and well-being [2, 3]. Generic HRQOL domain scores can be used to compare different diseases or other subgroups, assess interventions, and monitor individual patients [4, 5]. In addition, aggregates such as the Veterans RAND-36 physical and mental health summary scores provide higher-level summary information [6].

The Patient-Reported Outcomes Measurement Information System® (PROMIS®)-29 v2.1 is a state-of-the-science HRQOL profile measure [7]. The PROMIS-29 v2.1 assesses pain intensity using a single 0–10 numeric rating item and 7 health domains (physical function, fatigue, pain interference, depression, anxiety, ability to participate in social roles and activities, and sleep disturbance) using 4 polytomous (5 response categories) items per domain. If a study shows improvement on some scales and decrements in others, it can be difficult to draw an overall conclusion. For example, one treatment might look better than another in physical functioning, but a little worse in pain and anxiety, and not different in the ability to participate in social roles and activities. Is one treatment better than the other? To make concluding statements, it may help to summarize the multiple scale scores. The PROMIS-29 physical and mental health summary scores are weighted combinations of PROMIS-29 scale scores and are more reliable than domain scores and more likely to capture significant individual change [8, 9].

Wilson and Cleary [10] hypothesized a causal path from disease and treatment physiology to symptoms, then to functioning, next to general perceptions of health, and finally overall quality of life. General perceptions of health are assessed in PROMIS by 10 global health items: 5 overall rating items (physical function, fatigue, pain, emotional distress, and social health) and 5 general health perceptions items that cut across domains [11]. Four of the items are used for scoring the PROMIS global physical scale and 4 other items are used for the PROMIS global mental health scale. The PROMIS global physical health scale correlated most strongly with a computer adaptive test administration of the PROMIS physical function domain (r = 0.77) and the PROMIS global mental health scale with a computer adaptive test administration of the PROMIS depression and anxiety domains (r’s of − 0.72 and − 0.68, respectively) in a study of 1102 patients with ischemic and hemorrhagic strokes [12].

Schalet et al. [13] linked the PROMIS global health scales and the Veterans RAND-12 physical and mental health summary scores using data from 2025 adults in the Op4g internet panel. However, there are few comparisons of the PROMIS global health scales with the PROMIS-29 summary scores. Because these scores are part of the same measurement system, it might be assumed that they are comparable, but this is an empirical question. Neville et al. [14] found that the PROMIS global physical health and PROMIS-29 physical health T-scores were similar (45 and 47, respectively), but mental health scores differed (50 and 43, respectively) in a study of patients with severe COVID-19 6 months after a hospital intensive care admission.

While the PROMIS-29 physical health and mental health summary scores and the PROMIS global scales both putatively represent physical and mental health, the items and approach to deriving them differ substantially. Additional information about whether the PROMIS-29 physical and mental health summary scores and the PROMIS global health scales yield similar or different information is needed to provide guidance for their use in future research.

## Methods

### Samples

We analyzed data from Amazon’s Mechanical Turk (MTurk) and Ipsos’s KnowledgePanel (KP). As noted below, three longitudinal waves of data were analyzed from MTurk and one wave of data from KP. The PROMIS-29 v2.1 and PROMIS global health measures were administered to both samples. The analytic sample excluded those in the MTurk and KP samples who reported having one or both of two fake conditions (“Syndomitis” or “Chekalism”) included on the survey [15].

#### MTurk

Data were collected in 2021–2022 from the MTurk internet sample. Eligible study participants had to complete a minimum of 500 previous human intelligence tasks on MTurk with a successful completion rate of at least 95%. A sample of 5,804 adults completed general health questions on the baseline survey. A subset of the sample who on this survey reported currently having back pain (n = 1972) were asked to complete follow-up surveys: 1077 completed a 3-month survey and 845 a 6-month survey.

#### KP

The survey was also administered once in 2022 to a sample of 4060 adults from KP, an internet probability-based panel designed to represent the general U.S. population.

### Measures

The PROMIS-29 v2.1 and PROMIS global health items were administered. The PROMIS-29 physical health summary score is a combination of (in order of largest to smallest weight) physical function, pain, ability to participate in social roles and activities, fatigue, emotional distress, and sleep disturbance; the PROMIS-29 mental health summary score is a combination of (in order of largest to smallest weight) fatigue, emotional distress, ability to participate in social roles and activities, pain, sleep disturbance, and physical function.

The PROMIS global physical health score is estimated from 4 questions: (1) In general, how would you rate your physical health? (2) To what extent are you able to carry out your everyday physical activities? (3) How would you rate your pain on average? and (4) How would you rate your fatigue on average? The PROMIS global mental health score is estimated from 4 other questions: (1) In general, would you say your quality of life is… (2) In general, how would you rate your mental health? (3) In general, how would you rate your satisfaction with social activities and relationships? and (4) How often have you been bothered by emotional problems?

The physical and mental health scores for the PROMIS-29 and PROMIS global physical and mental health measures are scored on a T-score metric (mean = 50 and SD = 10 in the U.S. general population), with a higher score representing better health.

Nine retrospective change items were included in the 3-month follow-up of MTurk sample: All items used “Compared to three months ago” at the beginning. Eight of the items followed with: (1) In general, how is your physical functioning now? (2) In general, how is your ability to participate in social roles and activities now? (3) In general, how is your pain now? (4) In general, how is your fatigue now? (5) In general, how is your mood? (6) In general, how is your thinking (also known as cognition)? (7) In general, how is your sleep now? (8) how would you rate your health in general now? These items were administered using 5 response options (*Much better now than three months ago*; *Somewhat better now than three months ago*; *About the same*; *Somewhat worse now than three months ago*; *Much worse now than three months ago*). One retrospective change item included different response options: Compared to three months ago, is your back pain problem… (*Much worse*; *A little worse*; *About the same*; *A little better*; *Moderately better; Much better; Completely gone*). We scored each of the 9 items so that a higher score represented a more positive change in health.

### Human subjects protection

Study participants in both samples provided electronic consent upon starting the survey. All procedures were reviewed and approved by the research team's Institutional Review Board (RAND Human Subjects Research Committee FWA00003425; IRB00000051).

### Analysis plan

We estimate 3-month test–retest reliability estimates for the PROMIS-29 physical and mental health summary scores in the MTurk sample. Then, in the MTurk and KP samples, we provide mean PROMIS-29 physical and mental health summary scores and PROMIS global physical and mental health scores for 21 health conditions and for the overall sample at baseline. Based on prior estimates of the minimally important group difference [16, 17], we indicate where important differences exist between corresponding measures (PROMIS-29 versus PROMIS global)—that is, differences of 3 T-score points or more.

In addition, we estimate product-moment correlations between the PROMIS-29 v2.1 physical and mental health summary scores and the PROMIS global health physical and mental health scores in MTurk at baseline for the overall sample, and at 3 months later and 6 months later for those with back pain. We report results for the overall KP sample at the single administration. These are presented in the multitrait-multimethod (MTTM) product-moment correlation matrices among the PROMIS scales, with two “traits” (physical and mental health) measured by two methods (PROMIS-29 and PROMIS global). The MTMM matrices are analyzed to evaluate the construct validity of the measures [18]. Convergent validity is supported if the validity diagonal (“monotrait-heteromethod” correlations) consisting of correlations among measures of the same trait (e.g., physical health) assessed using different methods (e.g., PROMIS-29 v2.1 and PROMIS global health) are large. Discriminant validity is supported if: (1) correlations in the validity diagonal are larger than coefficients in the “heterotrait-heteromethod” and the “heterotrait-monomethod” triangles. We analyzed MTMM correlation matrices using the MTMM.EXE program [19]. In addition, we estimated correlations among changes in the PROMIS-29 and PROMIS global physical and mental health measures from baseline to 3 months later to see if changes over time in the two traits are similar for each method.

We also computed product-moment correlations between retrospective ratings of changes and changes in the PROMIS-29 and PROMIS global physical and mental health measures. Finally, we examined predictors of the PROMIS-29 and PROMIS global physical and mental health summary scores at the 3-month follow-up to better understand what may underlie any differences in the two sets of physical and mental health scores. We fit ordinary least square regression models that included baseline health, demographic characteristics (age, race/ethnicity, education), and indicators for 21 possible health conditions as right-hand side variables. We used Goodnight maximum R^2^ stepwise regression to identify significant independent variables [20]. This method assesses the effect of switching different variables on the total amount of variance explained. The first variable is selected which produces the largest R^2^ value. Once this variable is included in the model, a new variable is added that produces the largest incremental change in R^2^***.*** Variables are added (and/or deleted) at each step until the incremental change in the R^2^ no longer meets a previously determined level of significance ***(***p < 0.05) with the addition (and/or deletion) of any new variable, or a specified number of variables that maximize R^2^ have been entered.

## Results

### Sample characteristics

#### MTurk

At baseline, ages of those in the MTurk sample ranged from 18 to 89 with a median age of 36.5; 45% were female, 53% male, and 1% were transgender or did not identify as female, male, or transgender. Seventy percent were non-Hispanic White, 14% Hispanic, 9% non-Hispanic Black, 6% non-Hispanic Asians, and 1% were another race or multiracial. Eight percent reported a high school degree or less and 67% had a bachelor’s degree or higher.

#### KP

Ages of those in the KP sample ranged from 18 to 94 with a median age of 54; 50% were female, 50% male, and < 1% were transgender or did not identify as female, male, or transgender. Seventy percent were non-Hispanic White, 12% Hispanic, 10% non-Hispanic Black, and 8% non-Hispanic another race or multiracial. Thirty-three percent reported a high school degree or less, 26% some college or associate degree, and 41% had a bachelor’s degree or higher.

### Test–retest reliability for the PROMIS physical and mental health scores in MTurk

Test–retest product-moment correlations for the back pain sample (the only group for which we have longitudinal data) in MTurk for those who reported their back pain was the same at 3-months as at baseline were as follows: PROMIS-29 physical health (0.83), PROMIS-29 mental health (0.84), PROMIS global physical health (0.82), and PROMIS global mental health (0.83).

### PROMIS physical and mental health means by condition and overall sample

#### MTurk

The overall MTurk sample means (Table 1) ranged from 47 for global mental health to 50 for PROMIS-29 mental health. Mean scores by health conditions estimated by the PROMIS-29 and PROMIS global health scales were usually similar but 25% of the comparisons of the corresponding PROMIS-29 and global health scales differed by 3 or more T-score points (i.e., about 0.3 SD) with some higher and some lower. Specifically, the PROMIS global physical health scale was lower than the PROMIS-29 physical health summary score among respondents with anxiety and higher for heart attack, heart disease, and stroke. The PROMIS global mental health scale was 3 or more points lower than the PROMIS-29 mental health summary score for respondents overall as well as for those with anxiety or for those with depression, and it was higher for COPD, heart attack, heart disease, and stroke.

**Table 1:** Means scores on PROMIS Physical and mental health scores in MTurk

Condition (% of sample)	PROMIS-29 physical	Global physical	PROMIS-29 mental	Global mental
Anxiety (28%)	48	45↓	44	41↓
Depression (35%)	46	45	45	42↓
Trouble sleeping (14%)	47	45	44	43
COPD (5%)	42	43	43	46↑
Asthma (15%)	46	45	46	45
Arthritis (13%)	44	44	45	45
Cancer (5%)	45	45	46	46
Cholesterol (20%)	46	46	47	46
Diabetes (12%)	42	44	45	47
Angina (5%)	46	45	46	45
Hypertension (27%)	45	45	46	47
Heart Attack (5%)	41	44↑	43	49↑
Heart disease (5%)	41	44↑	43	49↑
Stroke (4%)	41	44↑	43	48↑
Back Pain (40%)	45	45	46	45
Sciatica (14%)	43	43	44	45
Neck pain (24%)	44	44	45	45
Dermatitis (11%)	46	44	44	43
Stomach trouble (21%)	45	44	44	43
Trouble seeing (14%)	44	44	44	44
Trouble hearing (8%)	44	44	44	45
Overall sample	49	48	50	47↓

Means for chronic back pain subgroup (24% of sample) were 44 on all 4 measures. Arrows indicate differences between corresponding PROMIS-29 and PROMIS global health scores of 3 or more T-score points. Overall sample n = 5804

#### KP

The overall KP sample means (Table 2) ranged from 49 for global physical health and global mental health to 53 for PROMIS-29 mental health and the differences were all in the same direction. The estimated PROMIS global mental health scores were lower than the PROMIS-29 mental health scale by 3 or more T-score points among respondents with anxiety, depression, high cholesterol, and the overall sample.

**Table 2:** Means scores on PROMIS physical and mental health scores in KP

Condition (% of sample)	PROMIS-29 physical	Global physical	PROMIS-29 mental	Global mental
Anxiety (20%)	47	45	45	42↓
Depression (20%)	46	44	45	42↓
Trouble sleeping (14%)	46	44	46	44
COPD (5%)	42	42	47	46
Asthma (13%)	48	46	49	47
Arthritis (30%)	45	45	50	48
Cancer (10%)	48	48	52	50
High Cholesterol (38%)	49	48	52	49↓
Diabetes (13%)	46	45	50	48
Angina (2%)	43	43	48	46
Hypertension (38%)	48	47	51	49
Heart Attack (3%)	45	45	50	50
Heart disease (6%)	46	45	51	50
Stroke (3%)	44	43	49	47
Back Pain (38%)	47	45	48	47
Sciatica (17%)	44	43	47	46
Neck pain (20%)	46	44	47	46
Dermatitis (10%)	47	46	48	46
Stomach trouble (15%)	46	44	46	45
Trouble seeing (14%)	45	44	47	45
Trouble hearing (15%)	47	46	50	48
Overall sample	51	49	53	49↓

Means for chronic back pain subgroup (21% of sample) were 45, 44, 48, and 46 for PROMIS-29 physical health, PROMIS global physical health, PROMIS-29 mental health, and PROMIS global mental health, respectively. Arrows indicate differences between corresponding PROMIS-29 and PROMIS global health scores of 3 or more T-score points. Overall sample n = 4060

### Correlations among PROMIS-29 and global physical and mental health scores

#### MTurk

MTMM correlations among the PROMIS-29 physical and mental health summary scores and the PROMIS global physical and mental health scales are shown in Table 3. The average convergent validity (monotrait-heteromethod) correlation was 0.63 and the average off-diagonal (heterotrait-monomethod and heterotrait-heteromethod) correlation was 0.57 at baseline in the MTurk sample. Only 3 of the 8 comparisons of validity diagonals with appropriate other correlations in the matrix were statistically significant in the hypothesized direction: that is, the correlation between the PROMIS-29 physical health summary score and PROMIS global physical health scores (r = 0.69) was statistically significantly (t = 54.34, p < 0.001) larger than the correlations of PROMIS-29 physical health summary score with the PROMIS global mental health (r = 0.21). It was also significantly (t = 12.54, p < 0.001) larger than the PROMIS global physical health with the PROMIS global mental health correlation (r = 0.55). In addition, the correlation of the PROMIS-29 mental health summary score and the PROMIS global mental health score (r = 0.56) was statistically significantly (t = 41.47, p < 0.001) larger than the correlation of the PROMIS-29 physical health summary score with PROMIS global mental health (r = 0.21). But three of the comparisons of validity diagonals were statistically significant in the wrong direction: the 0.69 physical health correlation versus the 0.74 correlation between PROMIS-29 mental health and PROMIS global physical health; the 0.56 mental health correlation versus the 0.68 correlation between PROMIS-29 physical and mental health, and the 0.74 correlation between PROMIS-29 mental and PROMIS global physical health. Further details of the MTMM analysis of these baseline correlations are given in the Supplemental Table 1.

**Table 3:** Multitrait-multimethod correlation matrix among PROMIS physical and mental health scores in MTurk and KP

MTurk	PROMIS-29		PROMIS global	
MTurk baseline	Physical	Mental	Physical	Mental
P-29 physical	1.00			
P-29 mental	0.68	1.00		
Global physical	**0.69**	0.74	1.00	
Global mental	0.21	**0.56**	0.55	1.00
MTurk 3-months				
P-29 physical	1.00			
P-29 mental	0.67	1.00		
Global physical	**0.76**	0.72	1.00	
Global mental	0.31	**0.63**	0.54	1.00
MTurk 6-Months				
P-29 physical	1.00			
P-29 mental	0.68	1.00		
Global physical	**0.81**	0.76	1.00	
Global mental	0.36	**0.69**	0.55	1.00
KnowledgePanel				
P-29 physical	1.00			
P-29 mental	0.68	1.00		
Global physical	**0.76**	0.75	1.00	
Global mental	0.37	**0.66**	0.60	1.00

MTurk baseline correlations in top third (n’s range from 5717–5846; median n = 5724), 3-month correlations (back pain subsample) in middle (n’s = 1031), and 6-month correlations (back pain subsample) in bottom third (n’s range from 826–827). KnowledgePanel (KP) sample n = 4060. Test–retest product-moment correlations between baseline and 3-months later were: 0.82 (PROMIS global physical health), 0.83 (PROMIS-29 physical health and PROMIS global mental health), and 0.84 (PROMIS-29 mental health)

The absolute value of the differences in percentile ranks for the physical health scores was 14 for KnowledgePanel and 16 MTurk at baseline. For the mental health scores the absolute value of the differences was 18 for KnowledgePanel and 22 for MTurk at baseline

Bolded entries are validity diagonals

At the 3-month follow-up, the average convergent validity correlation was 0.70 and the average off-diagonal correlation was 0.58. Six of the 8 comparisons of validity diagonals with appropriate other correlations in the matrix were statistically significant in the hypothesized direction but two of the correlations relating to mental health were in the wrong direction and one was statistically significant)—that is, the 0.63 correlation between the two mental health measures (Table 3) was significantly (t = − 4.58, p < 0.001) less than the 0.72 correlation between PROMIS global physical and mental health scores. At the 6-month follow-up, the average convergent validity correlation was 0.76 and the average off-diagonal correlation was 0.61. Six of the 8 comparisons of validity diagonals with appropriate other correlations in the matrix were statistically significant in the hypothesized direction, with one of the correlations significantly different in the wrong direction. In conclusion, there are noteworthy empirical differences in the two methods of measuring mental health.

#### KP

The average convergent validity correlation in the KP sample was 0.71 and the average off-diagonal correlation was 0.62. Only 5 of the 8 comparisons of validity diagonals with appropriate other correlations in the matrix were statistically significant in the hypothesized direction, with one of the correlations being significant in the wrong direction. The 0.66 correlation between the two mental health scores was significantly less (t = − 10.22, p < 0.001) than the 0.75 correlation between PROMIS-29 mental and PROMIS global physical health.

### Change from baseline to 3-months later in MTurk

The average change in the measures from baseline to 3 months later was minimal: − 1 T-score point for PROMIS-29 physical health, − 0.3 for PROMIS-29 mental health, 0.2 for global physical health, and 0.2 for global mental health. Correlations between the change from baseline to the 3-month follow-up on the physical health and mental health measures are shown in Table 4. Like what was seen for the correlations reported within the three waves of data collection, within method correlations between physical and mental health tended to be larger than between method correlations of the same trait (physical and mental health).

**Table 4:** Correlations of change between baseline and 3 months later in physical and mental health scores in the MTurk back pain subsample

	PROMIS-29		PROMIS global	
Change	Physical	Mental	Physical	Mental
P-29 Physical	1.00			
P-29 Mental	0.46	1.00		
Global physical	**0.41**	0.45	1.00	
Global mental	0.23	**0.36**	0.42	1.00

n = 1031

Bolded entries are validity diagonals

### Correlations with retrospective ratings of change in MTurk

The percentage of individuals reporting they were about the same on the retrospective change items was: 51% (mood), 55% (fatigue, back pain), 57% (sleep, health), 58% (pain overall), 61% (physical function), 64% (cognition), and 70% (social). Table 5 provides one-way ANOVA F-statistics and product-moment correlations between retrospective ratings of change and change in the physical and mental health scales. The largest correlation for each retrospective rating item is shown in bold: for 5 of the 9 retrospective items, it was with PROMIS global physical health, 2 for PROMIS-29 mental health, and 1 each for PROMIS-29 physical and PROMIS global mental health. Other than the back pain retrospective item, the ratio of F-statistics for the PROMIS global physical health scale compared to the PROMIS-29 physical health summary score ranged from 1.5 (pain) to 4.0 (health), indicating it was more sensitive to the retrospective items.

**Table 5:** Associations (F-statistics and product-moment correlations) of physical and mental health change between baseline and 3 months later on retrospective rating items in MTurk back pain subsample

Retrospective	P29 physical	Global physical	P29 mental	Global mental
Physical function (64%)	5.42 (0.14)	12.79 (**0.20**)	3.67 (0.11)	3.06 (0.09)
Social (70%)	3.77 (0.12)	7.05 (**0.15**)	6.37 (0.13)	2.69 (0.10)
Pain (61%)	7.63 (0.15)	11.48 (**0.20**)	7.67 (0.15)	4.15 (0.10)
Fatigue (55%)	2.84 (0.09)	7.01 (0.16)	10.97 (**0.18**)	2.32 (0.09)
Mood (51%)	2.44 (0.08)	5.50 (0.13)	6.82 (0.13)	6.77 (**0.16**)
Cognition (64%)	3.73 (0.10)	7.76 (**0.17**)	3.49 (0.10)	3.39 (0.09)
Sleep (57%)	6.49 (0.10)	10.72 (0.17)	12.18 (**0.18**)	5.96 (0.14)
Health (57%)	2.84 (0.10)	11.50 (**0.20**)	5.77 (0.13)	5.61 (0.13)
Back pain (55%)	6.43 (**0.18**)	5.46 (0.14)	5.51 (0.13)	2.17 (0.06)

n = 1031. P29 = PROMIS-29. Global = PROMIS global. Percentages in parentheses in the first column indicate those who reported they were the same on the retrospective change item. F-statistic from one-way ANOVA (product-moment correlations). Bold indicates largest correlation in the row

### Multivariate associations with physical and mental health 3-months post-baseline in MTurk

Significant variables and standardized betas from the regressions of 3-month physical and mental health measures, respectively, on baseline health, demographics, and medical conditions are given in Table 6. Not surprisingly, baseline health was by far the strongest predictor of health at 3 months post-baseline. None of the demographic variables were significantly uniquely associated with mental health measures. The health conditions significantly associated with the two mental health measures completely differed.

**Table 6:** Standardized regression coefficients for predicting PROMIS physical and mental health scores at 3-months by baseline health, demographics and conditions in MTurk back pain subsample

	PROMIS-29 physical health	PROMIS global physical health	PROMIS-29 mental health	PROMIS global mental health
Baseline Health	0.80	0.70	0.77	0.78
Hypertension	− 0.05			
Trouble sleeping	− 0.04	− 0.06		
Arthritis		− 0.07		
Depression		− 0.06	− 0.06	
Trouble hearing			− 0.05	
Heart disease			− 0.04	
Asthma			− 0.03	
Anxiety				− 0.06
Dermatitis				− 0.05
Stroke				− 0.03
Other race	− 0.03			
Adjusted R^2^:	68%	57%	67%	66%

Significant standardized regression coefficients are shown

## Discussion

The mean T-scores for the corresponding PROMIS-29 and PROMIS global physical and mental health scales were similar, but the PROMIS global mental health score was lower (worse mental health) than the PROMIS-29 mental health summary score by 3 T-score points in the MTurk sample and 4 points in the KP sample. In both samples, the lower PROMIS global mental health score than the PROMIS-29 mental health summary score was seen among those who reported that a doctor or other health professional told them they had anxiety (28% of the MTurk sample and 20% of the KP sample) or depression (35% of the MTurk sample and 20% of the KP sample). PROMIS-29 and PROMIS global mental health scores 6 months after intensive care for COVID-19 showed even larger differences (7 T-score points lower for PROMIS global mental health) than the current study [14]. So, the current study provides further evidence that the PROMIS global measure can yield lower mental health scores (indicating worse mental health) than the PROMIS-29 mental health summary score.

The correlations of 0.69–0.81 among physical health and 0.56–0.69 among mental health scales in this study are similar in magnitude to those reported by Schalet et al. [13] between the PROMIS global health scales and the Veterans RAND-12 physical and mental health scales (product-moment correlations of 0.69 between the physical health scales and 0.63 between the mental health scales). But the MTMM correlation matrices for the three survey administrations in MTurk and the single administration in KP, and the correlations among change in the measures between baseline and 3-months later in MTurk, showed that the PROMIS mental health measures correlated as highly with physical health as with the other mental health measure. Hence, this is the first study to evaluate and find a lack of discriminant validity for the PROMIS global mental health scale.

In contrast, correlations between the SF-12 version 2 physical component summary (PCS) and PROMIS global physical health scale (r = 0.78) and between the SF-12 version 2 mental component summary (MCS) and the PROMIS global mental health (r = 0.62) exceeded correlations between the SF-12 PCS and MCS (r = 0.26) and between the PROMIS global physical health and mental health scores (r = 0.55) in a sample of older adults in the New Zealand Health, Work and Retirement longitudinal study [21]. The authors concluded that the SF-12 PCS and PROMIS global physical health scale were similarly sensitive to hospital use and recurrent falls, but the SF-12 MCS was more sensitive to depression (CES-D score > 10) than the PROMIS global mental health scale. Schalet et al. [13] did not examine discriminant validity, but an MTMM matrix we created (see Supplemental Table 2) from that dataset supports discriminant validity for the physical health measures. Three of the four comparisons of the 0.62 validity diagonal correlation between the PROMIS global and VR-12 mental health scales support discriminant validity, but the 0.62 correlation was significantly smaller than the 0.69 correlation between the PROMIS global physical and mental scales (t = − 4.56, p < 0.001).

It is worth noting that discriminant validity findings for the SF-12/VR-12 MCS comparisons with the PROMIS global mental health scale are somewhat more favorable in part since the SF-12 and VR-12 PCS and MCS scores were created to be uncorrelated with one another [22, 23]. However, when the correlation between physical and mental health is estimated then noteworthy positive correlations between them have been observed. For example, product-moment correlations between physical and mental health factors at each of 3 years (baseline, 2-years post-baseline, and 4-years post-baseline) in the MOS ranged from 0.32 to 0.41 in the Medical Outcomes Study [24]. Similarly, a correlation of 0.53 between physical and mental health factors was reported in a study of 1053 older individuals (average age 64 years) sampled from an academic general medical clinic [25]. In addition, a correlation of 0.66 between RAND-36 physical and mental health was found in a sample of 255 females and 245 males stratified by age, race/ethnicity, and educational level to reflect the US population [26]. Finally, a correlation of 0.64 between the PROMIS global physical and mental health scales was observed in a recent study of 2,668 nonoperative patients at the time of their first visit to a multidisciplinary spine clinic [27]. This bolus of literature indicates that physical and mental health are positively correlated, and this can make it challenging to demonstrate discriminant validity when the methods of measure differ such as between the PROMIS-29 and the PROMIS global physical and mental health scores.

Correlations between mental health change scores and retrospective rating of change items in the MTurk sample were generally similar and small in magnitude, ranging from 0.09 (change in PROMIS global mental health with retrospective rating of change in cognition) to 0.18 (change in PROMIS-29 mental health summary score with retrospective rating of change in fatigue and with change in sleep). None of the correlations met the 0.371 level suggested for the use of anchors to estimate group-level minimally important differences [28]. This was in part because the majority (range of 51% for mood to 70% for social) reported on the retrospective items on the 3-month survey that they were about the same as 3 months ago, and the correlations were larger if those who did not change were excluded but they were still below the threshold (results not shown). In short, retrospective ratings and prospective change in PROMIS-29 and PROMIS global physical and mental health scores were only weakly associated with one another.

The regression models indicated that baseline health was by far the strongest predictor of the physical and mental health scales at the 3-month follow-up and only a few demographic and condition indicators were significantly uniquely predictive. There was one overlap in the conditions that predicted physical health (trouble sleeping) and the significant predictors of mental health differed. Depression was uniquely predictive of PROMIS-29 mental health while anxiety predicted the PROMIS global mental health scale score.

The results of this study indicate that conclusions about mental health in studies may differ based on whether the PROMIS-29 or PROMIS global mental health measure is used. Given the noteworthy difference in the PROMIS-29 mental health summary and the PROMIS global mental health scores, it is important to explore the reasons why in future research. While theoretically assessing the same construct, the measurement approach for the PROMIS-29 and PROMIS global health items is fundamentally different. The PROMIS-29 summary scores are weighted (factor scoring coefficients) combinations of PROMIS-29 domain scores while the PROMIS global mental health scale directly assesses mental health perceptions and is scored using item parameters from an IRT graded response model. When the PROMIS-29 is administered, a more nuanced and complete picture of HRQOL can be obtained by examining the 7 domain scores and the pain intensity item in addition to the physical and mental health summary scores. The 10 PROMIS global health items have the advantage of being brief, but the PROMIS-29 provides more detailed and rich information.

In conclusion, this study documents noteworthy differences in the PROMIS mental health summary scores estimated using a weighted combination of PROMIS-29 domain scores and the PROMIS 4-item global mental health scale. Investigations are needed to shed additional light on the implications of these differences and to provide guidance about the conditions for which one or the other scores (or use of both) is appropriate.

### Supplementary Information

Below is the link to the electronic supplementary material.

Supplementary file1 (DOCX 20 kb)

Supplementary file1 (DOCX 18 kb)

## Data Availability

The dataset analyzed for the current study is not publicly available due to the project being still in progress but are available from the second author on reasonable request.
