# Supplementary material for: Comparison of patient-reported outcomes measurement information system (PROMIS®)-29 and PROMIS global physical and mental health scores
Source: Qual Life Res. 2023 Dec 27;33(3):735–44. doi: 10.1007/s11136-023-03559-y (PMC10894145; doi:10.1007/s11136-023-03559-y)
Supplement: Supplementary file 1 — Supplementary file1 (DOCX 20 kb) [file 11136_2023_3559_MOESM1_ESM.docx]

**Supplemental Table 1. Multitrait-Multimethod Program**

Correlation Matrix Input Is As Follows:

MTURK PROMIS PHYSICAL MENTAL (BASELINE)

N = 5724; DFS = 5721

METHOD 1 2

TRAIT 1 2 1 2

---------------------------------------

1. 1.PHYSICAL 1.00

2.MENTAL .68 1.00

2. 1.PHYSICAL [.69] .74 1.00

2.MENTAL .21 [.56] .55 1.00

(Total Z = 1.48 Mean Z = .74)

Average convergent validity correlation is .629

Average off-diagonal correlation is .574

===================================================

Discriminant validity t-tests are as follows:

===================================================

Validity Coefficient = .69 for PHYSICAL

between Methods 1 & 2

Off-Diagonal Correlation

--------------------------

Method/Trait Method/Trait T P>|T| R R*

------------ ------------ ------- ----- ----- --------------------------

1/ MENTAL 1/ PHYSICAL 1.53 .126 .68 .74 (monomethod)

2 2 12.54* .000 .55 .21

2 1 54.34* .000 .21 .55 (heteromethod)

1 2 -7.45* .000 .74 .68

-----------------------------------------------

Number of successes: Monomethod 1/ 2

Heteromethod 1/ 2

===================================================

Validity Coefficient = .56 for MENTAL

between Methods 1 & 2

Off-Diagonal Correlation

--------------------------

Method/Trait Method/Trait T P>|T| R R*

------------ ------------ ------- ----- ----- -----------------------------------

1/ PHYSICAL 1/ MENTAL -10.71* .000 .68 .21 (monomethod)

2 2 1.30 .194 .55 .74

2 1 -21.48* .000 .74 .55 (heteromethod)

1 2 41.47* .000 .21 .68

-----------------------------------------------

Number of successes: Monomethod 0/ 2

Heteromethod 1/ 2

T = t-test recommended by Steiger (1980).

P>|T| = Probability of t, computed using STAT-SAK program

by Gerard E. Dallal and Owen (1965).

R = Correlation between different traits measured by either

different methods or same methods.

R* = Correlation between elements unique to the validity

coefficient & R above.

===================================================

SUMMARY: Number of Successes by Trait

===================================================

Trait Methods Success/Total

---------------------------------------------------

PHYSICAL 1 & 2 (Monomethod) 1/ 2

1 & 2 (Heteromethod) 1/ 2

MENTAL 1 & 2 (Monomethod) 0/ 2

1 & 2 (Heteromethod) 1/ 2

===================================================

TOTAL number of successes: Monomethod 1/ 4

Heteromethod 2/ 4

===================================================
