# Supplementary material for: Comparison of patient-reported outcomes measurement information system (PROMIS®)-29 and PROMIS global physical and mental health scores
Source: Qual Life Res. 2023 Dec 27;33(3):735–44. doi: 10.1007/s11136-023-03559-y (PMC10894145; doi:10.1007/s11136-023-03559-y)
Supplement: Supplementary file 2 — Supplementary file1 (DOCX 18 kb) [file 11136_2023_3559_MOESM2_ESM.docx]

**Supplemental Table 2. Multitrait-Multimethod Correlation Matrix Among PROMIS Physical and Mental Health Scores in Op4G Internet Sample**

VR-12 PROMIS Global Health

|  | Physical | Mental | Physical | Mental |
| --- | --- | --- | --- | --- |
| VR-12 Physical Health | 1.00 |  |  |  |
| VR-12 Mental Health | 0.23 | 1.00 |  |  |
| PROMIS Global Physical Health | **0.71** | 0.48 | 1.00 |  |
| PROMIS Global Mental Health | 0.41 | **0.62** | 0.69 | 1.00 |

Note: n = 2014. VR-12 = Veterans RAND-12 Health Survey.

Bolded entries are validity diagonals.
